# In TFIIH the Arch domain of XPD is mechanistically essential for transcription and DNA repair

**DOI:** 10.1038/s41467-020-15241-9

**Published:** 2020-04-03

**Authors:** Stefan Peissert, Florian Sauer, Daniel B. Grabarczyk, Cathy Braun, Gudrun Sander, Arnaud Poterszman, Jean-Marc Egly, Jochen Kuper, Caroline Kisker

**Affiliations:** 1 Rudolf Virchow Center for Experimental Biomedicine, Institute for Structural Biology, University of Würzburg, 97080 Würzburg, Germany; 2 Institut de Génétique et de Biologie Moléculaire et Cellulaire Illkirch Cedex, C.U., Strasbourg, France; 3 Centre National de la Recherche Scientifique, UMR7104, 67404 Illkirch, France; 4 Institut National de la Santé et de la Recherche Médicale, U1258, 67404 Illkirch, France; 5 Université de Strasbourg, 67404 Illkirch, France; 6 Comprehensive Cancer Center Mainfranken, Würzburg, Germany

**Keywords:** DNA, Enzyme mechanisms

## Abstract

The XPD helicase is a central component of the general transcription factor TFIIH which plays major roles in transcription and nucleotide excision repair (NER). Here we present the high-resolution crystal structure of the Arch domain of XPD with its interaction partner MAT1, a central component of the CDK activating kinase complex. The analysis of the interface led to the identification of amino acid residues that are crucial for the MAT1-XPD interaction. More importantly, mutagenesis of the Arch domain revealed that these residues are essential for the regulation of (i) NER activity by either impairing XPD helicase activity or the interaction of XPD with XPG; (ii) the phosphorylation of the RNA polymerase II and RNA synthesis. Our results reveal how MAT1 shields these functionally important residues thereby providing insights into how XPD is regulated by MAT1 and defining the Arch domain as a major mechanistic player within the XPD scaffold.

## Abstract (web summary)

XPD is part of the TFIIH complex which plays major roles in transcription initiation and nucleotide excision repair (NER). Here the authors present a high-resolution crystal structure of the XPD-MAT1 interface and dissect the role of this interface in transcription and NER.

## Introduction

**Fig. 1:**
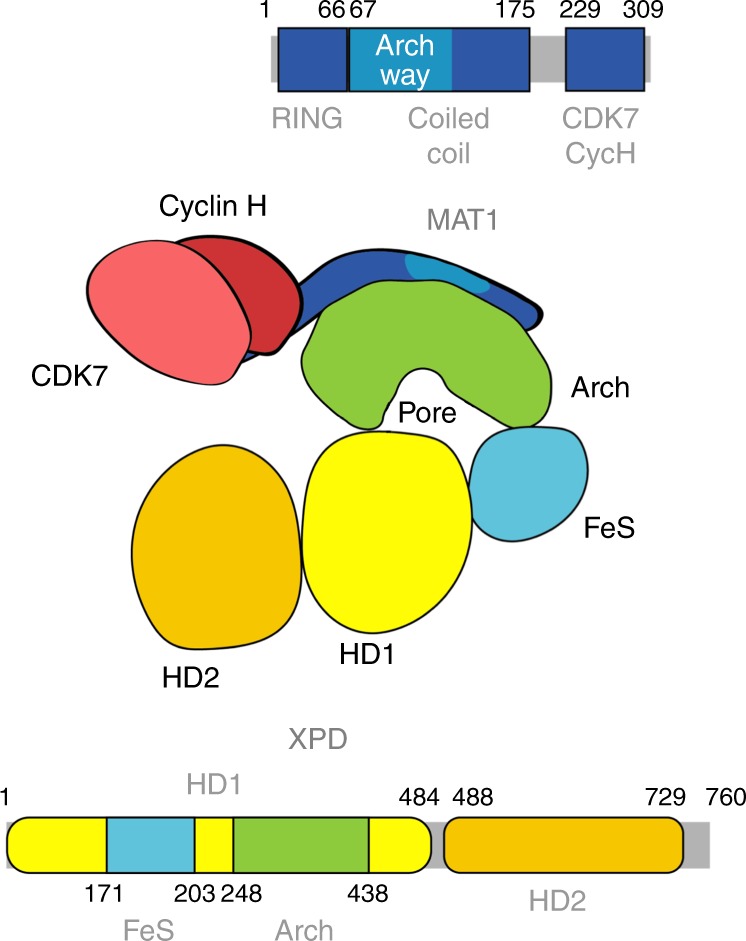
Cartoon representation of human XPD and human CAK. The domain architecture of XPD and MAT1 within the linear sequence of the two proteins is shown. The location of the different domains is indicated and labeled. A schematic of the CAK–XPD complex utilizing the same colors as in the linear sequence cartoon is shown with Cyclin H and CDK7 in two different reds.

The super family 2 (SF2) helicase XPD is a 5′–3′ helicase and a founding member of the group of iron sulfur cluster (FeS) containing helicases^1^. These helicases all share a common fold that consists of two RecA like motor domains (HD1 and HD2), the FeS domain and an additional Arch domain. This basic scaffold has been first structurally described for archaeal XPDs^2–4^. One of the most enigmatic features of XPD helicases is a pore like structure that is built by the closure of the Arch domain and the FeS domain both originating from HD1 (Fig. 1). The path of the translocated single stranded DNA (ssDNA) on XPD has been identified by a combination of structural and biochemical methods. It extends from a high affinity binding site in HD2 to HD1. The DNA then threads through the pore thereby closely passing the FeS and finally reaches a second high affinity binding site located at the distal end of the pore^5,6^. To permit ssDNA to pass through the pore, the Arch domain has to disengage from the FeS domain leading to a pore opening while the protein interacts with the DNA^7,8^. A connection between the dynamics of the pore, the ATPase activity, and the translocation cycle has been proposed^9,10^ but the mechanism remains poorly understood. For eukaryotic XPD the basic scaffold has been extended to accommodate the diverse tasks it has to fulfill as an essential member of the general transcription factor II H (TFIIH) and beyond^11,12^.

TFIIH is a multi-protein complex that is involved in RNA polymerase II (Pol II) mediated transcription and nucleotide excision DNA repair (NER)^13^. Its importance is highlighted by mutations in individual TFIIH subunits which lead to the severe human hereditary diseases xeroderma pigmentosum, trichothiodystrophy, and Cockayne Syndrome^12,14^. TFIIH consists of ten subunits of which p8, p34, p44, p52, p62, XPB, and XPD belong to the core complex (core TFIIH) whereas MAT1, CDK7, and Cyclin H comprise an additional module, the CDK activating kinase complex (CAK). Core TFIIH and CAK are bridged via the MAT1 subunit that links XPD to CDK7/Cyclin H^15,16^. MAT1 is a modular protein that contains an N-terminal RING domain (residues 1–66), a proposed coiled coil structure in its middle region (residues 67–175) and a hydrophobic region at the C-terminus (residues 229–309) that interacts with CDK7/Cyclin H (Fig. 1). The interaction between core TFIIH and CAK via XPD plays a pivotal role in the processes of transcription and NER. In transcription, the link between core TFIIH and CAK is crucial for the effective phosphorylation of the C-terminal domain of Pol II and the phosphorylation of nuclear receptors upon transactivation through CDK7^17^. In NER, however, the CAK module has to be removed via an XPA-dependent mechanism for completion of the damage verification and excision process^18^, which is well in line with the observation that core TFIIH DNA binding and activity is decreased in the presence of CAK^15,19^. XPD, the main CAK interaction partner, has to fulfill completely different tasks in the processes of NER and transcription. In transcription, XPD anchors and positions the CAK module, thereby only being important as a scaffolding protein, whereas its full enzymatic activity is indispensable for NER^20^ to accomplish unwinding of the DNA around the lesion and to participate in damage verification^21–23^.

The main anchor point for the CAK complex on core TFIIH is the Arch domain of XPD^15^. Recent cryo-EM structures of TFIIH localized the coiled coil domain of MAT1 on the Arch domain of XPD^24,25^. These studies also confirmed that the overall scaffold of eukaryotic XPD and its domain architecture is highly comparable to the high-resolution structures of XPD from archaea. However, how the inhibition of XPD via MAT1 is accomplished could not be deduced. Here, we present the high-resolution crystal structure of the Arch domain of human XPD in complex with the central domain of MAT1 providing an atomic resolution model of this important regulatory interaction. Based on the structure we identified functionally important residues in the Arch domain of XPD that are vital for its interaction with MAT1, and thereby the phosphorylation of RNA polymerase II and RNA synthesis but also for its helicase activity and NER progression. Our analysis reveals that MAT1 regulates XPD not only by reducing the DNA binding capacity of XPD but also shields residues essential for XPD helicase activity and DNA repair thereby rendering XPD completely inactive.

## Results

### The molecular interface of XPD and MAT1

We solved the crystal structure of the complex containing the central domain of human MAT1 (66–141) and the Arch domain of XPD (245–439). The structure was solved by SAD phasing (see Methods section for details). Native crystals diffracted up to 2.07 Å and belong to space group P2_1_2_1_2_1_. The final model was refined to R-factors of 0.192/0.227 (R_work_/R_free_) containing two Arch/MAT1 heterodimers in the asymmetric unit (Supplementary Table 1). Within MAT1 residues 66–129 are visible, the last 12 residues seem to be disordered. For the Arch domain residues 246–439 can be observed in both monomers with residues 294–316 and 291–316 being disordered in monomer A and B, respectively (Supplementary Fig. 1a).

**Fig. 2:**
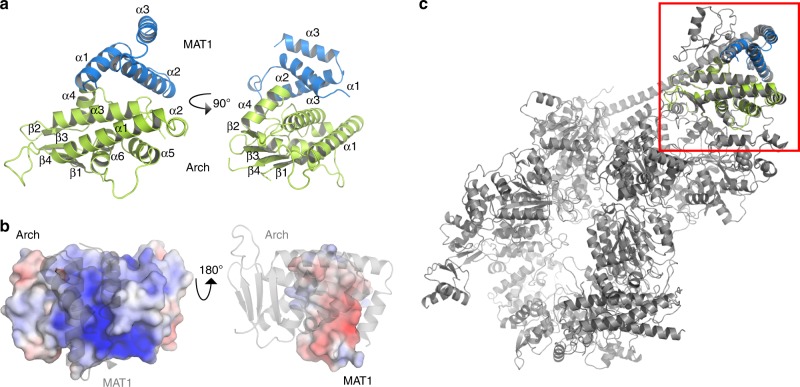
Structure of the human MAT1–Arch domain complex. **a** Overall fold of the MAT1 (blue) and Arch domain (green) complex in two orientations rotated 90° around the *y*-axis. The secondary structure elements are labeled. **b** Molecular surface representation with electrostatic potential mapping of either the Arch (left) domain or MAT1 (right). Blue denotes positively charged regions and red negatively charged regions. The surface was generated and plotted using the apbs plugin of pymol. The borders for plotting the potential were set to −6 for negative charge and +6 for positive charge. The respective binding partner is shown in cartoon representation (gray, 50% transparency). **c** Overall structure of TFIIH (pdb code 6nmi) in cartoon representation. The red square shows the superposition of our human Arch/MAT1 structure with TFIIH (cartoon representation, color coding as in A, core TFIIH is depicted in gray).

The two heterodimers are nearly identical as indicated by an rmsd of 0.48 Å. Notably, the two molecules in the asymmetric unit show a domain swap between the Arch domain monomers where the two central β-strands of the Arch domain base, which is composed of an antiparallel 4 β-strand motif, are exchanged (Supplementary Fig. 1a). Since the domain swap can be viewed as a crystallographic artifact, we generated one Arch/MAT1 heterodimer with the central β-sheet being derived from the other molecule in the asymmetric unit for all subsequent analyses (Fig. 2a). MAT1 contains three α-helices which form a helical-bundle: two (α1 and α2) helices interact with the Arch domain and the third (α3) packs above them and thereby stabilizes the fold (Fig. 2a). The Arch domain comprises a total of six α-helices and engages with MAT1 via α3 and the loop structure between α4 and α5. The overall surface area covered upon complex formation encompasses 727 Å^2^ and mainly involves charged interactions. The interface is thus characterized by a significant charge complementarity between the negatively charged MAT1 and the positively charged Arch domain (Fig. 2b). Importantly, our high resolution structure can be superimposed with the most recent medium resolution TFIIH cryo-EM structure (pdb code 6nmi) yielding an rmsd of 1.3 Å, thus confirming that the same architecture is observed in the overall TFIIH complex^24,25^ further supporting its functional relevance (Fig. 2c).

### Biochemical characterization of the Arch/MAT1 interface

**Fig. 3:**
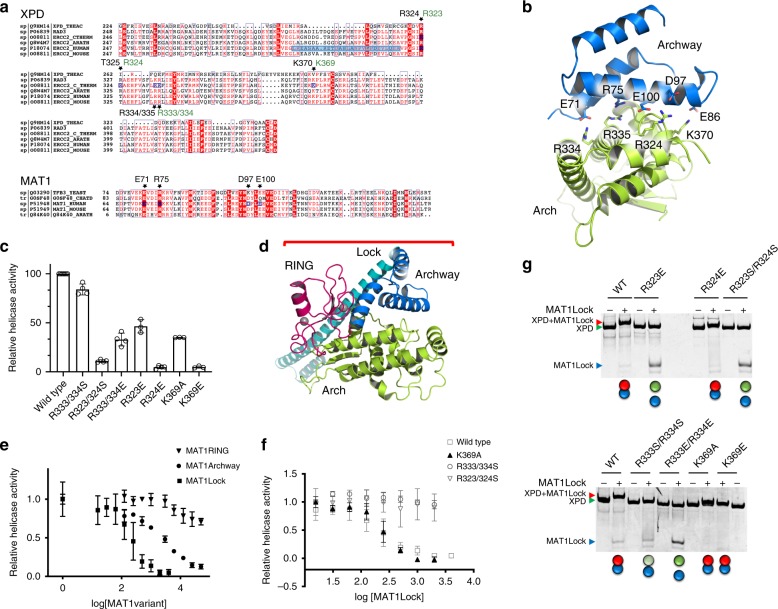
Functional mutagenesis studies on ctXPD and ctMAT1Lock. **a** Sequence alignment of the Arch domain of XPD and the coiled coil region of MAT1 from different organisms (order of appearance: *T. acidophilum*, *S. cerervisiae*, *C. thermophilus*, *A. thaliana*, *H. sapiens*, and *M. musculus*; uniprot code indicated). Targeted residues are marked with a star and are labeled in black for the human and in green for the *C. thermophilum* proteins. The plug region in human XPD is marked in blue. **b** Cartoon representation of the human Arch domain MAT1 complex with the residues chosen for functional mutagenesis studies highlighted in stick representation; color coding is the same as in Fig. 2a. **c** Helicase activity of 250 nM wild-type ctXPD or its variants in the presence of 250 nM ctp44 measured using a 5′OH substrate. The helicase activity of the wild-type complex was set to 100%. Average values and SD are shown and were derived from two biological replicates combined in at least three technical replicates. **d** Model of MAT1 engaged with the Arch domain of XPD based on pdb code 6nmi. The MAT1RING is shown in magenta, MAT1Archway in blue, Arch in green, and in cyan we have labeled the extension of MAT1 visible in the 6nmi structure. **e** ctXPD helicase inhibition in the presence of the ctMAT1 constructs ctMAT1RING, ctMAT1Archway and ctMAT1Lock. Average values and SD are shown and were derived from at least three technical replicates. **f** Helicase activity of wild-type ctXPD and ctXPD variants in the presence of increasing concentrations of MAT1Lock. Average values and SD are shown and were derived from at least three technical replicates. **g** Native PAGE analysis of the ctXPD ctMAT1Lock interaction. The green and red circles below the gels indicate whether complex formation was observed. Green represents free ctXPD and blue free MAT1Lock (lightgreen indicates partial complex formation), whereas red is used for ctXPD when ctXPD and ctMAT1Lock form a complex (also indicated by the contacting circles). Source data are provided as a Source Data file.

To analyze the importance of the Arch/MAT1 interface we pursued biochemical studies utilizing homologous proteins from the fungus *Chaetomium thermophilum*, which are more readily amenable to biochemical analysis and were validated to be functionally highly comparable to their human orthologues^20^. We focused on strictly conserved residues that are involved in central interactions between MAT1 and the Arch domain (Fig. 3a, b). As indicated by the sequence alignment in Fig. 3a, the two arginines, R323 and R324, in *C. thermophilum* XPD (ctXPD) correspond to R324 in human XPD which is located in a loop region just adjacent to α3 and interacts with residues E100 and D97 from MAT1 via a salt bridge. Curiously, in budding yeast, *Arabidopsis thaliana* and *C. thermophilum* there seems to be a conserved twin arginine motif at this position, whereas the second arginine corresponds to a threonine in human and mouse XPD. In addition, another twin arginine motif (R334/R335) is located in the center of α3 that corresponds to R333/R334 in ctXPD. In our structure R334/R335 interact with E71 and R75 from MAT1; R335 stacks with R75 whereas R334 and E71 engage in a salt bridge. Lastly, we also targeted K369, corresponding to K370 in human XPD which interacts with E86 from MAT1 (see also Supplementary Fig. 1b).

All generated ctXPD variants (R323S/R324S, R323E, R324E, R333S/R334S, R333E/R334E, K369A, and K369E) expressed like the wild-type protein and showed no significant melting point alteration in thermal unfolding assays, indicating no structural impairment of the generated variants (Supplementary Fig. 2a, Supplementary Methods). We first investigated the helicase activity of ctXPD and its variants (Fig. 3c and Table 1, see Methods section). The importance of the R333/R334 pair for XPD helicase activity is only moderate since the reverse charge variant reduces the activity to 32.9% whereas the serine exchange (R333S/R334S) has nearly no effect. To our surprise, however, the R323S/R324S variant showed a significant decrease with a reduction to 10.9% activity. In contrast, the single R323E variant displayed about 46% residual activity, indicating only a minor influence on ctXPD activity, whereas the charge reversal in the subsequent residue (R324E) impaired the helicase almost completely (4.7% residual activity). These results suggest that of the R323/R324 pair R324 is of critical importance for ctXPD helicase activity. Lastly, we investigated the K369A/E variants. Both display a significant decrease with 35.0% and 4.8% residual activity, respectively. K324E and K369E thus showed the strongest inhibition of helicase activity among all tested variants.

**Table 1:** Kinetic parameters of ctXPD variants.

Variant	Helicase, %	DNA-dependent ATPase activity, µmol ATP l^−1^ min^−1^	DNA binding, *K*_D_, nM	DNA binding, *K*_D_, nM +MAT1
Wild type	100.0	42 ± 1.1	66 ± 1.1	379 ± 1.4
R323S/R324S	10.9 ± 1.4	34 ± 0.8	51 ± 1.2	31 ± 1.1
R323E	46.4 ± 6.8	n. p.	41 ± 1.1	n. p.
R324E	4.7 ± 1.5	n. p.	72 ± 1.2	n. p.
R333S/R334S	84.0 ± 5.6	51 ± 1.4	154 ± 1.1	128 ± 1.2
R333E/R334E	32.9 ± 6.5	n. p.	108 ± 1.1	n. p.
K369A	35.0 ± 0.1	n. p.	n. p.	n. p.
K369E	4.8 ± 1.1	n. p.	55 ± 1.1	n. p.

*K*_D_ values were determined using the “Sigmoidal, 4PL, *X* is log(concentration)” function in GraphPad Prism.

n.p. not performed.

As XPD helicase activity is negatively regulated by CAK^15,26^, we investigated which parts of MAT1, the central component of the CAK (Fig. 1), are required for the association with XPD and lead to helicase inhibition. We generated three different ctMAT1 versions; ctMAT1RING (residues 1–83) comprising the RING domain, ctMAT1Archway reflecting the crystallization construct (residues 83–157 in ctMAT1), and the combination of RING and Archway which we called ctMAT1Lock (residues 1–157, see Fig. 3d). To analyze the inhibitory effect of these three constructs on ctXPD we investigated their influence on helicase activity in titration experiments. To our surprise the ctMAT1Archway construct only led to a mild inhibition with an IC_50_ value of around 3 µM and the ctMAT1RING alone showed no discernable inhibition under our experimental conditions. Only the combined ctMAT1Lock variant displayed a high affinity inhibition with an IC_50_ of 220 nM (Fig. 3e, Table 2). We further investigated the interaction between ctXPD and ctMAT1 fragments via native polyacrylamide gel electrophoresis (PAGE) analysis and observed here as well that the ctMAT1Archway variant displayed a reduced affinity toward ctXPD compared to the ctMAT1Lock variant as indicated by the reduced complex formation at similar concentrations (84% unbound ctMAT1Archway to 12.8% unbound ctMAT1Lock, Supplementary Fig. 2c). The RING domain of ctMAT1 thus seems to influence the affinity toward ctXPD. To rule out a possible artifact, we performed similar experiments with the human proteins. Pull-down experiments with immobilized human MAT1 fragments showed that the MAT1Archway (residues 66–141) was able to retain full length human XPD. As observed with the *C. thermophilum* proteins, the interaction of this minimal domain from MAT1 with XPD was weak. Experiments conducted with MAT1Lock (residues 1–141) confirmed that the RING finger domain stabilizes the association of MAT1 with XPD (Supplementary Fig. 3a). We therefore conducted all further ctMAT1 inhibition experiments with the ctMAT1Lock construct.

**Table 2:** Relative helicase activity inhibited by ctMAT1.

Variant	MAT1Lock IC50 (nM)	MAT1Archway IC50 (nM)	MAT1RING IC50 (nM)
Wild type	220 ± 1.2	2992 ± 1.4	no fit
R333S/R334S	no fit	n. p.	n. p.
R323S/R324S	no fit	n. p.	n. p.
K369A	263 ± 1.1	n. p.	n. p.

IC50 values were determined using the “Sigmoidal, 4PL, *X* is log(concentration)” function in GraphPad Prism”.

No fit: data could not be fitted, since no saturation in binding could be reached under the experimental conditions used.

To assess whether the variants in the Arch domain of ctXPD interfere with ctMAT1 binding we chose only those variants that still displayed significant helicase activity (Fig. 3c) and analyzed them toward inhibition by ctMAT1Lock. The K369A variant showed the same inhibition pattern by ctMAT1Lock, as observed for wild-type XPD (Fig. 3f and Table 2). This residue therefore seems to be important for ctXPD helicase activity but neglectable for the ctMAT1 interaction. In contrast, neither the R333S/R334S variant that retained wild-type helicase activity nor the R323S/R324S variant that displayed 12.9% residual activity, were inhibited by ctMAT1Lock, suggesting that these mutations directly affect the ctMAT1 ctXPD interaction (Fig. 3f and Table 2). Importantly, these results show that single amino acid exchanges within the ctMAT1Archway/Arch interface succeeded to completely disrupt the interaction between ctXPD and ctMAT1Lock thus implying that the influence of the MAT1 RING domain toward binding to XPD is not essential.

We next investigated the interaction profile of the ctXPD variants toward ctMAT1Lock using native PAGE analysis (Fig. 3g). Here the R333S/R334S variant led to partial complex formation in the presence of high ctMAT1Lock concentrations, which can most likely be attributed to the high protein concentrations used in the native PAGE experiment compared to those used in the helicase assays. The R333E/R334E variant was unable to interact with ctMAT1Lock suggesting that this motif is mainly involved in mediating the interaction with MAT1 and only assumes some minor mechanistic influence on helicase activity. The K369A/E variants clearly interact with ctMAT1Lock in the native PAGE analysis. Finally, the double R323S/R324S and the single R323E variant are unable to interact with ctMAT1Lock whereas the R324E variant supports complex formation comparable to the wild-type proteins.

### Mechanism of helicase inhibition

**Fig. 4:**
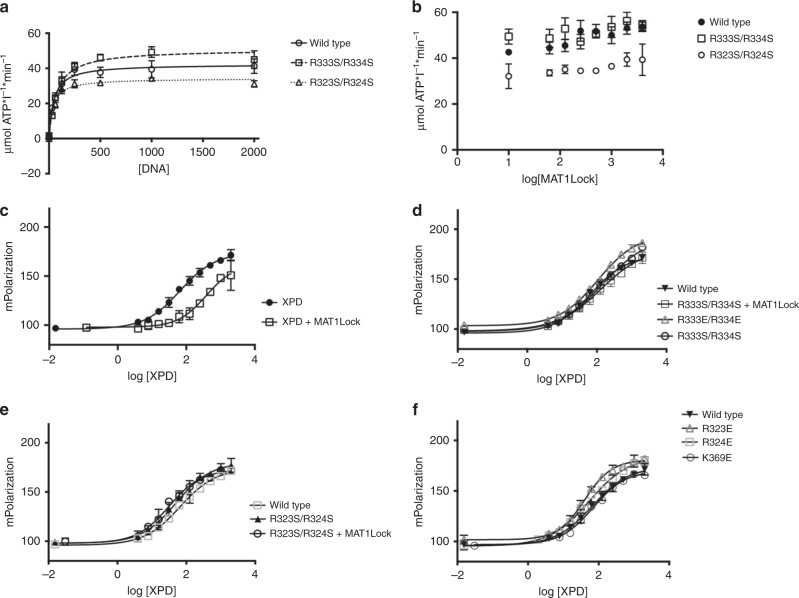
DNA dependent ATPase activity and DNA binding parameters of ctXPD variants. **a** ssDNA dependent ATPase activities of ctXPD and selected variants using increasing amounts of DNA. **b** ctMAT1Lock influence on ssDNA induced ATPase of wild-type ctXPD and ctXPD variants. **c**–**f** Fluorescence anisotropy measurements utilizing wild-type ctXPD and ctXPD variants in the presence or absence of equimolar amounts of ctMAT1Lock and a splayed duplex DNA as bait. Average values and SD are shown and were derived from two biological replicates combined in at least three technical replicates.

To decipher the cause for the reduced helicase activity, we analyzed the ATPase activity of wild-type ctXPD, the R323S/R324S and R333S/R334S variants in the presence of ctp44, titrating increasing amounts of ssDNA to the reactions. Surprisingly, ssDNA dependent ATPase activity seems to be almost unaffected by either of the variants as indicated by apparent *V*_max_ values that range from 42 µmol ATP l^−1^ min^−1^ for wild-type ctXPD, 51 µmol ATP l^−1^ min^−1^ for the R333S/R334S, and 34 µmol ATP l^−1^ min^−1^ for the R323S/R324S variant (Fig. 4a and Table 1). Only the R323S/R324S variant seems to exhibit a mild effect on ATPase activity. Our data therefore indicate that the lack of helicase activity is not caused by a diminished ATPase activity of ctXPD. We then investigated whether ctMAT1Lock is able to inhibit the ATPase activity in a concentration dependent way. Neither wild-type ctXPD nor the two variants showed any inhibitory effect induced by ctMAT1Lock even at a 16-fold molar excess (Fig. 4b), supporting our observation that the ATPase activity is not targeted by MAT1 inhibition or by any of the Arch domain variants. We then analyzed the DNA binding affinity of wild-type ctXPD in the presence and absence of ctMAT1Lock toward a splayed duplex DNA using fluorescence anisotropy (Fig. 4c and Table 1). ctXPD interacts with this DNA substrate with a *K*_d_ of 66 nM that is reduced to 379 nM by the addition of equimolar amounts of ctMAT1Lock, indicating that MAT1 impacts the DNA binding capacity of ctXPD. This is in line with previous data which showed that CAK influences DNA binding of XPD^15^. The R333S/R334S variant showed a decreased *K*_d_ of 154 nM and the addition of ctMAT1Lock does not alter this significantly (*K*_d_ 128 nM). The R333E/R334E variant has a *K*_d_ of 108 nM which is in a similar range. Since all three variants show a mild elevation in the *K*_d_ this could indicate a small but measurable impact on DNA binding by these variants (Fig. 4d and Table 1) which is not increased in the presence of ctMATLock1 due to the abolished interaction of the two proteins. The affinity of the R323S/R324S variant toward the splayed fork substrate is 51 nM and corresponds well to wild-type ctXPD and due to the inability to interact with MAT1 it is not altered by the addition of ctMAT1Lock (31 nM) (Fig. 4e and Table 1). Finally, we analyzed the three reverse charge variants R323E, R324E, and K369E. All these variants showed no impairment in DNA binding with *K*_d_ values of 41, 72, and 54 nM, respectively (Fig. 4f and Table 1). Our data thus clearly show that wild-type ctXPD is affected by the presence of ctMAT1Lock toward its ability to interact with DNA. In contrast, the variants display a mixed behavior even in the absence of ctMAT1Lock. Whereas the R333E/R334E and the R333S/R334S variants display a slightly reduced affinity toward DNA, the R323E, R324E, and K369E variants are not affected thus suggesting that residues which are involved in the interaction with ctMAT1Lock could also be involved in DNA binding.

### The XPD Arch/MAT1 interface in transcription and NER

**Fig. 5:**
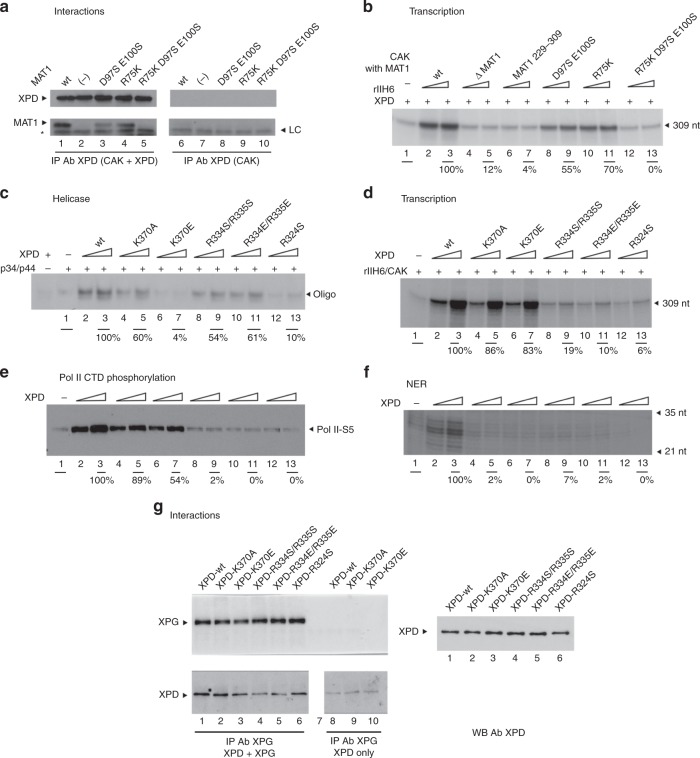
Functional analysis of human XPD and MAT1 variants. **a** XPD/CAK interactions analyzed using a pull-down assay with immobilized XPD (Lanes 1–5). Control extract (for details see Method section; lanes 6–10). MAT1 and XPD were detected using specific antibodies. The asterisk marks the light chain of the antibody (LC). **b** In vitro transcription activity assay of wild-type MAT1 and its variants. Purified CAK lacking MAT1 (∆ MAT1) or containing its variants (100 and 250 ng) were added to a reconstituted system containing core-TFIIH lacking XPD (rIIH6) (250 ng) and XPD (200 ng). Transcripts were analyzed by electrophoresis followed by autoradiography (309 nt run-off transcript) and was normalized to that of wild-type CAK/MAT1. Numbers correspond to the average values obtained for the two quantities of CAK tested. **c** XPD Helicase assay. Increasing amounts of XPD wild type and variants (100 and 200 ng) were added to a 5′-strand extension probe (for details see Method section; lanes 2–13). Controls were performed in absence of p34/p44 or XPD as indicated. The reaction was analyzed by electrophoresis followed by autoradiography and helicase activity (unwound oligonucleotide, black arrow) was normalized to that of wild-type XPD. The average activity estimated from three independent experiments (*n* = 3, mean, ±sd) using the highest XPD concentration is indicated in Table 3, whereas the panel shows a representative experiment. **d** In vitro transcription activities of reconstituted TFIIH containing XPD variants (see Methods section for details). The size of the transcript is indicated by the black arrow and the transcription activity estimated using the highest XPD concentration is indicated below the autoradiography. The average activity estimated from four independent experiments (two independent sets of reactions performed with the two XPD concentrations, *n* = 4, mean ± sd) is indicated in Table 3, whereas the panel shows a representative experiment. Lane labels of **c** and **d** apply also to **e** and **f**, respectively. **e** Phosphorylation activities of the TFIIH complexes analyzed by immunoblotting using an antibody directed against the phosphorylated form of serine 5 from the CTD heptad repeat. **f** In vitro NER activity of reconstituted TFIIH containing XPD variants. XPD wild type or variants (100 and 200 ng, see Method section for details). The NER product is visible between the 35 and 21 nt limits. **g** Pull-down assay with XPG and wild-type XPD as well as XPD variants. Lanes 1–6 present the pulldown experiments where XPG was immobilized with an anti-XPG antibody. For control experiments an extract expressing the DsRed protein instead of human XPG was used (lanes 8–10). XPD and XPG were detected by Western Blot using monoclonal antibodies. The XPD loading controls of lanes 1–6 are shown on the right side of the panel.

Based on the results described above, we set out to examine the effect of our variants in transcription and NER. We designed full-length human MAT1 variants encompassing D97S/E100S, R75K, and the combination of the two variants yielding R75K/D97S/E100S. We purified the corresponding CAK complexes (Supplementary Fig. 3b) and performed pull-down experiments. Incubation with wild type and the purified CAK variants clearly showed that wild-type CAK associates with XPD (Fig. 5a, lane 1). The R75K variant showed a slightly reduced interaction with MAT1 while the double variant D97S/E100S and the triple variant R75K/D97S/E100S decreased or entirely abolished the interaction with MAT1 (Fig. 5a, lanes 3 and 5). In experiments where the human Arch domain was co-expressed with MAT1Lock (residues 1–141) in *E. coli*, a soluble Arch/MAT1 complex was obtained while the Arch domain in isolation did not yield soluble protein (Supplementary Fig. 4, compare C1 and C2). The MAT1Lock variants E71S, R75K, and D97S/E100S showed a significantly reduced interaction with XPD in this assay whereas the E86S variant still supported complex formation (Supplementary Fig. 4, lanes 1–4), thus displaying a stronger phenotype than observed in the pull-down experiments. For further characterization, the purified CAK complex variants together with core-TFIIH were analyzed for their capacity to stimulate transcription using an in vitro reconstitution assay. The single variants D97S/E100S and R75K only mildly affected the transcriptional activity (55% and 70% residual activity, respectively; Fig. 5b, lanes 8–11) whereas the triple variant R75K/D97/E100S was unable to stimulate RNA synthesis (compare lane 1 with lanes 12 and 13), comparable to the MAT1 variant 229–309 that only comprises the interaction region for CDK7/Cyclin H or a CAK lacking MAT1 (lanes 4–7). These results thus underline the key role of the Arch/MAT1 interaction in gene expression.

For further investigation we introduced the ctXPD variants in human XPD. As described above, human XPD only contains a single arginine in position 324 whereas the following residue is a threonine (corresponds to R324 in ctXPD). We therefore introduced the R324S (corresponds to R323 in ctXPD) variant into human XPD (R324S). For the remaining ctXPD residues R333/R334 and K369 we generated the corresponding human XPD variants R334S/R335S, R334E/R335E, K370A, and K370E. We first investigated the helicase activity. XPD wild-type readily unwinds a 5′ overhang substrate in the presence of p34/p44 (Fig. 5c, lanes 2 and 3; Table 3; please note that the raw data for Table 3 can be found in Supplementary Tables 2 and 3). The K370A variant shows a lower activity of 38% (Fig. 5c, lanes 4 and 5; Table 3), whereas the inverted charge of the K370E variant led to a high decrease yielding only 6% activity (Fig. 5c, compare lanes 6 and 7 to lane 1; Table 3). The two variants R334S/R335S and R334E/R335E both display substantial remaining activity with 45% and 56%, respectively (Fig. 5c, lanes 8–11, Table 3). Finally, the R324S variant shows a strong impairment in helicase function with 10% activity (Fig. 5c, lanes 12 and 13, Table 3). These observations are well in line with the ctXPD data thus indicating a high mechanistic consistency between the two helicases (for comparison see Fig. 3c and Table 1).

**Table 3:** Summary of human XPD activities.

XPD	Helicase (%)	NER	Transcription (%)	RNA Pol Phos
wt	100	++	100	++
K370A	38 ± 20	–	91 ± 13	+
K370E	9 ± 5	–	84 ± 7	+
R334S/R334S	45 ± 13	–	25 ± 12	−
R334E/R334E	56 ± 17	–	26 ± 17	−
R324S	10 ± 3	–	8 ± 6	−

Raw data for Table 3 is provided as Supplementary Tables 2 and 3.

Based on the knowledge that XPD is essential for both, transcription and NER^12,20^, we examined how the Arch domain variants would affect these two TFIIH activities. In a transcription assay, we analyzed the XPD variants for their ability to recruit CAK into a preformed rIIH6 complex that includes all core TFIIH subunits. Wild-type XPD (100%), the K370A (91%), and the K370E (84%) variants were able to successfully reconstitute in vitro transcription. (Fig. 5d, lanes 2–7; Table 3) whereas the R334S/R335S, R334E/R335E (25% and 26%, respectively) and the R324S variants led to a severe reduction in RNA synthesis (8%, Fig. 5d, lanes 8–13; Table 3), thus providing further support for the impaired XPD MAT1 interaction.

We next investigated whether the defect in in vitro transcription correlates with a failure in the RNA Pol II CTD phosphorylation (as a marker of ongoing transcription). All the complexes that show impaired transcription also display a highly reduced phosphorylation activity (Fig. 5e, lanes 8–13), while the wild type and the K370 variant complexes supported CTD phosphorylation (Fig. 5e, lanes 2–7; Table 3). We finally tested the XPD variants in a dual-incision NER assay. Wild-type XPD successfully removed the damaged oligonucleotide (Fig. 5f, lanes 2–3) from the template. Surprisingly, the NER assays showed that none of the XPD variant-containing TFIIH complexes supported damaged oligonucleotide removal (Fig. 5f, lanes 4–13; Table 3), indicating a total loss of NER function for all the XPD variants regardless of whether they still retain helicase activity or not. To further elucidate the nature of the NER impairment we investigated whether the variants are still able to interact with the endonuclease XPG which has been shown to interact with XPD^15^. We performed pull down experiments using XPG as bait and analyzed the interaction with XPD and its variants. Figure 5g shows that the K370E, and R324S variants and wild-type XPD are able to interact with XPG while the R334S/R335S and R334E/R335E variants show a significantly impaired interaction.

## Discussion

**Fig. 6:**
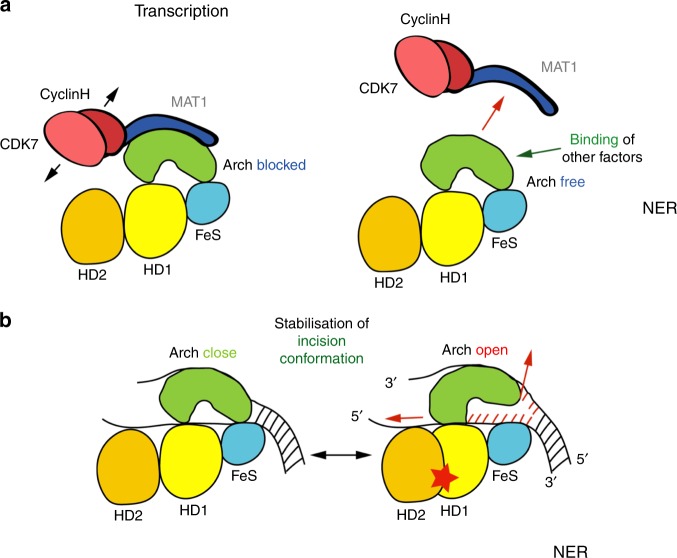
Involvement of the Arch domain in the helicase mechanism. **a** Schematic representation of XPD and CAK in transcription and NER. **b** Cartoon representation of the suggested Arch domain dynamics related to ATP hydrolysis thereby aiding in strand separation.

We solved the high-resolution structure of a complex formed between the Arch domain of XPD and its direct interaction partner MAT1. The molecular details of the XPD/MAT1 interface enabled us to probe residues that are essential for the interaction of both proteins and further analyze their impact on XPD activity. We identified a twin arginine motif at position 333/334 that is mainly involved in MAT1 interactions. Mutation of this motif abrogated complex formation completely (Fig. 3f, g) but interestingly displayed only a moderate impact on the helicase function and also a slight decrease in DNA binding affinity (Fig. 3c and Fig. 4d). The corresponding interacting residues in MAT1 are E71 and R75 which both show a reduced Arch/MAT1 interaction when mutated to a serine or a lysine (E71S and R75K) in the co-expression analysis (Supplementary Fig. 4) thus further supporting the importance of these residues for complex formation. However, when full length MAT1 was used, R75K was not sufficient to significantly prevent the interaction as indicated by the pull-down assays and only a slight reduction in transcription activity (Fig. 5a, b). The MAT1 D97S/E100S double variant which shows a serious impairment toward its ability to interact with XPD, led to a decrease of transcriptional activity likely due to the absence of a functional CAK core TFIIH interaction. Consequently, the mutation of R324 in XPD that interacts with the D97/E100 pair also leads to a significant impairment in the transcription assays (Fig. 5d). In contrast, the K370E (K369 in ctXPD) variant, which displays a severely impaired helicase activity in both, the human and the *C. thermophilum* system (Figs. 3c and 5c), is able to interact with MAT1; the resulting TFIIH is thus able to phosphorylate RNA pol II and RNA synthesis can be initiated (Figs. 3g and 5d, e). This clearly demonstrates the key role of the Arch/MAT1 interface to position the CAK for accurate transcription initiation. Although our experiments show that the RING domain is of importance for the stabilization of the XPD–MAT1 interaction (Fig. 3e; Supplementary Figs. 2c and 3a) this effect can be neglected in the context of the interface analyzed in this study, since mutations in the XPD Arch/MAT1 interface are sufficient to abrogate productive interactions in isolation but also in the context of the transcription machinery (Fig. 5b, d, e). The Arch domain of XPD thus serves as the major anchoring point to mediate CAK function, thereby building the bridge between core TFIIH and CAK and providing sufficient flexibility for CDK7 to phosphorylate the CTD and at the same time ensuring that XPD is maintained in an inactive state (Fig. 6a). The crucial Arch/MAT1 interaction is further supported by the recent cryo-EM structure of a PIC that shows that MAT1Lock is interacting with TFIIE via the MAT1 RING domain, thereby further stabilizing the conformation in the PIC^24^. However, this additional interaction is not sufficient to rescue the loss of interaction in the essential Arch/MAT1 interface.

Intriguingly, we identified residues that are critical for helicase activity within the Arch/MAT1 interface, namely K370 and R324. K370 (K369 in ctXPD) is highly important for helicase activity (Figs. 3c and 5c) but assumes no major role in the MAT1 interaction. R324 or the corresponding twin arginine motif R323/R324 in ctXPD, assumes a dual role. On the one hand R324 is vital for the XPD–MAT1 interaction but it is also highly important for the helicase activity of XPD. Interestingly, it seems that this dual function is split in other organisms which also harbor a twin arginine motif in this position, with the first arginine (R323 in *C. thermophilum*) being responsible for the MAT1 interaction and the second (R324) for helicase activity (Fig. 3c, f, g). The lack of helicase activity cannot be explained by the loss of ATPase activity, since the R323S/R324S variant displays almost wild type like ATPase function. Similarly, MAT1Lock inhibits the helicase activity very efficiently but the ATPase activity is not altered. Since the ATPase cycle is unaffected by the presence of MAT1 or the introduction of a mutation at position 324, all the elements necessary for the ATPase cycle must be intact. This is not surprising considering that the ATPase function of XPD is mainly mediated by motifs in HD1 and HD2, where the major ssDNA binding sites in XPD have been identified^5,6,8^ and are not in close proximity to the Arch/MAT1 interface (Fig. 6b). Our results thus suggest that an initial DNA interaction in the presence of MAT1 is possible but does not lead to helicase activity due to Arch domain blocking. This is further supported by a recent study describing the DNA translocation mechanism of DinG, the *E. coli* homolog of XPD^27^. In this study the ssDNA fragment extends toward the Arch domain of DinG spanning both HD1 and HD2 which is also thought to be a prerogative for triggering the ATPase activity. Since the ATPase activity is not altered, the question remains how the Arch domain affects the helicase activity? A recent cryo-EM study proposed a plug region in the Arch domain (residues 292–318, Fig. 3a, Supplementary Fig. 5a) that could potentially block the way of the ssDNA through the pore and thereby regulate helicase activity^28^. This region is in close vicinity to the MAT1 binding site. Indeed, R324 is located at the very end of the plug region but the major part of the plug does not interact with MAT1 and thus a direct influence from MAT1 on the overall stability of the entire region seems unlikely (Supplementary Fig. 5a). In addition, the observation that R324 does not only provide an interaction site with MAT1 but is also highly important for helicase and NER activity, supports a more mechanistic function of this residue. Furthermore, our analysis of K370 which is not interacting with the proposed plug region but, when mutated also severely affects the helicase and NER activity, further supports the importance of the positively charged region as the major regulator of XPD activity.

**Fig. 7:**
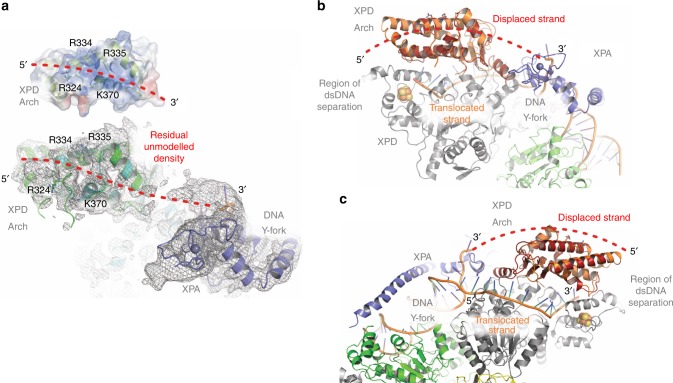
The path of the non-translocating DNA strand in TFIIH. **a** Comparison of the proposed DNA binding region to the positively charged area on the Arch domain (upper panel) and the same orientation in the TFIIH complex (pdb code 6ro4). The trajectory of ssDNA as observed in the TFIIH structure can be readily extended toward the proposed region in the Arch domain (red dashed line) and is decorated with residual cryo-EM density that could indicate the presence of the remaining 16 bases of the DNA substrate used in the study. The contour level of the EM map is shown at 3.5*σ*. The Arch domain of XPD from the cryo-EM structure (pdb code 6ro4, cyan) was superpositioned with our crystal structure (green). Both structures are highly homologous and the crucial residues are located in similar positions. Please note that R324 could not be modeled in the EM structure. XPA is shown in purple and the 3′ as well as the 5′ end of the DNA strand are indicated. **b**, **c** Two views of the cryo EM structure of Kokic et al. (pdb code 6ro4) to illustrate the path of the dsDNA and ssDNA in the complex. XPD is shown in gray for the helicase and FeS domains, the superimposed Arch domains are shown in light and dark brown, XPA in purple and XPB in green. The extended path of the ssDNA is shown as a red dashed line. View in **c** is rotated 180° around the *y*-axis as compared to **b**.

This positively charged surface patch of the Arch domain (Figs. 2b and 7a) contains R324, R334/R335, and K370 that may reflect a mechanistically important minor DNA interaction site on the Arch domain. Blocking the complete site via MAT1 leads to an almost fivefold decrease in DNA binding. In the absence of MAT1, however, this effect can only be observed to a small extent since the major high affinity ssDNA binding sites are still intact^5,6,8,20^ and may therefore compensate for the loss of ssDNA affinity on the Arch domain. The Arch domain is proposed to be a dynamic element in XPD that can open and close in a manner which might be linked to ATP hydrolysis^8–10^. Kokic et al.^28^ show the path of the translocating ssDNA strand in XPD indicating that it threads through the XPD pore within TFIIH (Fig. 7b, c) as has been shown earlier for DinG and archaeal XPD. Combined with the observation that the DinG Arch domain rotates and opens during the ATP driven translocation cycle^27^ it is very likely that human XPD utilizes a similar mechanism. In the TFIIH cryo EM structure of Kokic et al. a Y-fork DNA substrate was used. In the structure, only the first 4 of the 20 bases of the displaced strand were shown. Interestingly, however, the trajectory from this displaced DNA strand leads to the positively charged area of the Arch domain identified as being important in our study. This trajectory is decorated with fragments of cryo-EM density that could represent the loosely bound ssDNA strand of the remaining 16 unmodeled bases thus supporting our suggested model for the position of the non-translocating DNA strand (Fig. 7a). We therefore propose that once the MAT1 interaction has been released, the Arch domain can actively engage with DNA during the translocation cycle, via its positively charged residues on the surface (R324 and K370). Importantly, in this model the Arch domain does not engage with the translocating strand but with the displaced strand. Strand separation in XPD has to be achieved in the area where the iron sulfur cluster domain and the Arch domain are located, however, the exact elements involved in strand separation still remain unknown (Fig. 7). Due to the ATP dependent motions during the translocase cycle, the Arch domain could aid in the separation of the two DNA strands when it switches between the open and closed state by engaging the displaced strand while XPD is translocating in a 5′–3′ direction on the translocating strand (Figs. 6b and 7b, c). This model is well in line with our data and the structural and biochemical data currently available and provides a different perspective on the Arch domain of XPD. It defines the Arch domain not only as a structural highly important entity that mediates crucial protein-protein interactions but also as a functionally substantial element that is mechanistically important for XPDs helicase activity. Due to this dual role, the interaction with MAT1 via the Arch domain inactivates functionally important residues by shielding them from the environment. Since the Arch domain is an element that is recurrent in the entire XPD helicase family, the proposed mechanism could also be employed by other family members. The archaeal XPD structure revealed a positively charged region on the Arch domain similar to eukaryotic XPD^2^ (Supplementary Fig. 5b) suggesting that the mechanistic function of the Arch domain has been most likely acquired early in evolution. To test whether other XPD helicase family members employ a similar mechanism we built a homology model of DDX11 from *C. thermophilum* and investigated a potentially positively charged region that is located in the same area as the positively charged region in ctXPD (Supplementary Fig. 5c–e, Supplementary methods). The K427E/K428E variant displayed slightly altered DNA binding parameters (*K*_d_ 30.8/50.8 nM, wild type to K427E/K428E) and showed a decrease in helicase activity, thus suggesting a similar role of these residues in DDX11.

Intriguingly, we observed that all XPD Arch domain variants are severely impaired in their NER activity in vitro (Fig. 5f). Variants that show impaired helicase activity are consequently unable to support repair^20,29^. However, even the K370A, R334S/R335S, and R334E/R335E variants that still display helicase activity, fail to be active in NER. The K370A variant retains 38% of wild-type helicase activity but is inactive in NER (Fig. 5f). Since the XPD K370E variant is completely inactive as a helicase, one could speculate that the exchange to an alanine, although it is not detrimental for helicase activity, might affect the ability of XPD to stabilize a repair competent DNA conformation (during or after lesion verification) in order to promote subsequent incision. Similarly, the R334/R335 variants could also assume a phenotype that slightly impairs the helicase but severely affects DNA stabilization which is subsequently necessary for the incision cascade (Fig. 6a, b). However, since these residues have already been implicated in protein–protein interactions and do not seem to be crucial for XPD helicase activity (in contrast to K370), it is tempting to speculate that this motif could be a potential target site for additional NER factors. The site occupied by both residues would only be accessible once CAK removal is achieved, and could then be occupied by other factors, such as XPG, to promote DNA incision (Fig. 6a). Since it was shown that XPG stabilizes TFIIH^30^ and a deletion of the ARCH domain partially impairs the XPD/XPG association^16^ we investigated the XPG interaction. Indeed, pull down experiments with the R334S/R335S and R334E/R335E variants show impaired complex formation and further substantiate this hypothesis.

In summary, our analysis of the Arch/MAT1 interface provides significant insights toward the importance of the Arch domain element in the XPD scaffold. Our data suggest that the Arch domain may be intimately involved in the strand separation process, thereby mediating helicase activity. This important role of the Arch domain is most likely conserved in the XPD helicase family, unraveling detailed mechanistic insights that improve our understanding how the highly important helicases XPD, DDX11, FANCJ, and RTEL accomplish substrate unwinding. With respect to XPD the Arch domain has to fulfill a dual function since it is required for transcription and represents the essential pivotal anchor for CAK function whereas in NER it is important for helicase function and aids toward recruiting XPG to coordinate successful damage removal.

## Methods

### Mutagenesis, expression, and purification

The coding sequences of human MAT1 (66–141) and XPD-Arch (245–439) were amplified by polymerase chain reaction from cDNA and inserted between the 3C protease cleavage site and BamHI restriction site of the pETM-22 (MAT1; EMBL vector collection) or pCDF-22 (XPD-Arch; in house vector carrying the same T7 expression cassette as pETM-22) vectors by the sequence and ligation independent cloning method (SLIC)^31^. Both constructs harbor an N-terminal thioredoxin–hexa-histidine tag which is cleavable by the HRV14-3C protease. The genes encoding full-length ctXPD, ctp44 (1–285), and ctMAT1 (1–84, 83–157, 1–157, and 1–248) were cloned from a cDNA library from *C. thermophilum* (provided by Ed Hurt). CtXPD and ctp44 were cloned into the pBADM11 and pETM11 vectors using SLIC, (EMBL-Heidelberg), respectively. CtXPD mutants were generated using the Quick-Change site-directed mutagenesis kit (Stratagene). The reactions were carried out as suggested by the manufacturer’s instructions. All variants were verified by double-stranded sequencing.

The MAT1 Arch domain complex was co-expressed in BL21star (DE3) cells (Invitrogen) carrying the pRARE2 plasmid (Novagen). Cells were grown in TB medium supplemented with 50 µg/ml streptomycin and kanamycin and 34 µg/ml chloramphenicol at 37 °C until they reached an OD_600_ of 2.5. Protein expression was induced by the addition of isopropyl-β-thiogalactopyranoside (IPTG) to a final concentration of 0.3 mM. After 12 h expression at 20 °C, the cells were harvested, resuspended in lysis buffer (50 mM Hepes pH 8.0, 300 mM NaCl, 1 mM TCEP supplemented with 1 tablet of Roche complete EDTA free protease inhibitor cocktail per 250 ml of cell lysate and DNAseI) and lysed using a mechanical cell disruptor (Microfluidics). After clarification of the lysate by centrifugation (35,000 × *g* for 1 h at 4 °C), the supernatant was twice applied to a gravity flow Ni-IDA column (Macherey and Nagel). The column was washed with 20 CV wash buffer (50 mM Hepes pH 8.0, 1 M NaCl) and bound proteins were eluted by the addition of 2 × 2 CV elution buffer (50 mM Hepes pH 8.0, 300 mM NaCl, 400 mM Imidazole). For removal of the N-terminal thioredoxin-hexa histidine tag from both complex partners, 1 mg HRV14-3C protease was added to the eluate and the protein was dialyzed over night against 20 mM Hepes pH 8.0, 300 mM NaCl, 14 mM β-Mercaptoethanol. The dialysate was concentrated using Amicon ultra centrifugal filters (Merck Millipore) and applied to a Superdex 200 16/60 pg column (GE healthcare) equilibrated with 20 mM Hepes, pH 8.0, 150 mM NaCl, 1 mM TCEP. The complex containing fractions were pooled, concentrated and reapplied to a Superdex 200 10/300 GL column (GE healthcare). The purified complex was then concentrated to a final concentration of 2.5 mg/ml (assuming a 1:1 Arch: MAT1 stoichiometry), flash frozen in liquid nitrogen and stored at −80 °C until further usage.

ctXPD wild type and the variants were expressed as N-terminally His-tagged proteins in *Escherichia coli* ArcticExpress (DE3)-RIL cells (Agilent). Cells were grown in TB medium at 37 °C until they reached an OD_600_ of 0.6–0.8. Expression was started with the addition of 0.05% l-arabinose and performed at 11 °C for 20 h. ctp44 (1–285) was expressed as N-terminally His-tagged protein in *E. coli* BL21-CodonPlus (DE3)-RIL cells (Stratagene). Cells were grown as described for ctXPD and expression was started by adding 0.1 mM IPTG at and performed at 14 °C for 18 h. ctXPD and ctp44 were purified to homogeneity by metal affinity chromatography (Ni-IDA, Macherey and Nagel) as described previously^20^ followed by size exclusion chromatography (SEC) (20 mM HEPES pH 7.5, 200 mM NaCl) and an additional anion exchange chromatography (AEC) step in the case of ctXPD. AEC was performed using a MonoQ 5/50 GL column (GE Healthcare) with 20 mM HEPES pH 7.5, 50 mM NaCl, and 1 mM TCEP as loading buffer and the same buffer containing 1 M NaCl was used for elution applying a gradient of 0–60% buffer B using 50 column volumes. The final buffer after AEC was 20 mM HEPES pH 7.5, 250 mM NaCl, and 1 mM TCEP. The proteins were concentrated to at least 5 mg/ml based on their calculated extinction coefficient using ProtParam (SwissProt) and then flash frozen for storage at −80 °C. ctMAT1 constructs were recombinantly expressed as N-terminally thioredoxin-His-tagged fusion proteins. Overexpression was carried out in *E. coli* BL21(DE3) star (Invitrogen) pRARE2 cells (Novagen) in TB-medium by addition of 0.1 mM IPTG for 18 h at 18 °C. The protein was purified by affinity chromatography (Ni-IDA, Macherey-Nagel) and the tag was cleaved off overnight utilizing the HRV-14 3C protease prior to SEC which was performed as described for ctXPD.

### Determination of the XPD Arch/MAT1 structure

Prior to crystallization, the thawed Arch/MAT1 complex (2.5 mg/ml) was stored at 4 °C for 12–20 h until the protein solution became turbid. The solution was then clarified by centrifugation (1 h at 25,000 × *g* and 4 °C) and the complex was crystallized at 20 °C using the hanging drop vapor diffusion method by mixing 2 µl of the complex solution with 2 µl of the crystallization buffer (100 mM Tris-HCl pH 8.5, 25% w/v PEG8000). Crystals appeared after 1–2 days and reached their maximum size after approximately 1 week. For data collection, crystals were briefly dipped into cryo buffer (100 mM Tris-HCl pH 8.5, 22% v/v PEG400) and then flash frozen in liquid nitrogen.

For the collection of anomalous data, crystals were incubated in derivatization/cryo buffer (100 mM Tris-HCl pH 8.5, 22% v/v PEG400, 5 mM K_2_IrCl_6_) for 5 min and then flash frozen in liquid nitrogen. Diffraction data were collected at ESRF beamlines ID23-2 (K_2_IrCl_6_ derivatized crystals) and ID29 (native crystals), integrated with XDS^32^ and scaled with Aimless^33^.

The structure was solved by the single wavelength anomalous dispersion method (SAD) exploiting the anomalous signal of a single ordered IrCl_6_^2−^ ion using the CRANK2 phasing pipeline^34^. After substructure determination using CRUNCH2^35^, phasing with REFMAC^36^ and density modification with Parrot^37^, an initial model composed of 475 amino acids in 6 fragments was automatically built with Buccaneer^38^. This model was then used to solve the structure by molecular replacement against native data extending to a resolution of 2.07 Å using PHASER^39^. The model was completed by iterative cycles of manual building with COOT^40^ and refinement of atomic positions, B-factors and TLS parameter in PHENIX^41^ (Supplementary Table 1).

### In vitro DNA dependent ATPase activity assay

ctXPD ATPase activity was measured utilizing an in vitro ATPase assay in which ATP consumption is coupled to the oxidation of NADH via pyruvate kinase and lactate dehydrogenase activities. Activities were measured at 30 °C in 100 µl solution, containing 1.5 U pyruvate kinase, 1.9 U lactate dehydrogenase, 2 mM phosphoenolpyruvate, and 0.3 mM NADH, 50 mM KCl, 5 mM MgCl_2_, 1 mM TCEP, and 20 mM HEPES (pH 7.5). The assay was carried out under saturating concentrations of ATP (2 mM) using ctXPD wild type, variants and ctp44 at a concentration of 250 nM. ssDNA (5′-TGACAGCTATGACCATGATTACGAATTGCTTGGAATCCTGACGAACTGTAG-3′) was added at final concentrations ranging from 31 nM to 2 µM as indicated. Studies including MAT1 were performed with equimolar amounts of ctXPD, ctp44, and ssDNA (250 nM) in the presence of increasing MAT1 concentrations. The mix of all reagents, with the exception of ATP, was preincubated at 30 °C until a stable base line was achieved. Enzyme catalysis was initiated by the addition of ATP. The activity profiles were measured at 340 nm using a Flourostar Optima (BMG labtech) plate reader. Reactions were followed until total NADH consumption was reached, which usually occurred within 10 min for wild-type ctXPD. Initial velocities were recorded and ATP consumption was determined using the molar extinction coefficient of NADH. The measurements were carried out in triplicates and with at least two different protein batches and mean values are plotted with their associated SD.

### In vitro helicase assay

Helicase activity was analyzed utilizing a fluorescence-based helicase assay. We used a 5′ overhang substrate with a cy3 label at the 3′ end of the translocated strand (5′-AGCTACCATGCCTGCACGAATTAAGCAATTCGTAATCATGGTCATAGC-3′-cy3, red color denotes ssDNA overhang) and a dabcyl modification on the 5′ end of the opposite strand (Dabcyl-5′- GCTATGACCATGATTACGAATTGCTT-3′). Assays were carried out in 20 mM HEPES pH 7.5, 50 mM KCl, 5 mM MgCl_2_, and 1 mM TCEP. DNA was used at concentrations of 16–1000 nM as indicated for the DNA activation experiments. Since maximum activity was observed already at a DNA concentration of 250 nM (Supplementary Fig. 2b), we used these parameters to elucidate the influence of the ctXPD variants on helicase activity. Helicase activity of wild-type ctXPD and its variants was measured at equimolar concentrations of ctXPD, ctp44, and 5′ overhang substrate (250 nM). The mix of all reagents, with the exception of ATP, was preincubated at 30 °C until a stable base line was achieved. The reaction was subsequently started with the addition of 2 mM ATP. Live Kinetic measurements were recorded with a Flourostar Optima plate reader (BMG labtech). Fluorescence was detected at an excitation wavelength of 550 nm (slid width, 2 nm) and an emission wavelength of 570 nm (slid width, 2 nm). Initial velocities of the kinetic data curves were fitted with the MARS software package (BMG labtech) and represent the averages of at least three different reactions and two independent protein batches and mean values are plotted with their associated SD.

### Native polyacrylamide gel electrophoresis (native PAGE)

Binding of ctXPD wild type and ctXPD variants to ctMAT1Lock was analyzed using 12% acrylamide gels. The gels were cast with 12.5 mM Tris acetate pH 6.9 and electrophoresis was performed in 12.5 mM Tris/96 mM glycine (0.5× Tris–glycine) running buffer. Prior to loading, the samples containing 10 µM of each protein—unless indicated otherwise—were incubated on ice for 30 min. 5 µl of the sample was supplemented with 1 µl of loading dye (50% glycerol, 0.1% Ponceau S) and loaded onto the gel. Electrophoresis was performed at 100 V and 4 °C for 100 min. The samples’ buffer composition was equal throughout all lanes and comprised 20 mM HEPES pH 7.5, 50 mM KCl, 5 mM MgCl_2_, and 1 mM TCEP.

### Fluorescence anisotropy

DNA binding was analyzed by fluorescence anisotropy employing a splayed duplex DNA with a cy3 label Cy3-5′- AGCTACCATGCCTGCACGAATTAAGCAATTCGTAATCATGGTCATAGC-3′ and 5′-GCTATGACCATGATTACGAATTGCTTGGAATCCTGACGAACTGTAG-3′ (red color denotes ssDNA region). Assays were carried out in 20 mM HEPES pH 7.5, 50 mM KCl, 5 mM MgCl_2_, 1 mM TCEP, and 5 nM DNA at room temperature. Wild-type ctXPD and its variants were used at concentrations of 2–2000 nM as indicated. After mixing, the reaction was incubated for 5 min prior to recording. Fluorescence was detected at an excitation wavelength of 540 nm and an emission wavelength of 590 nm with a Floustar Optima plate reader (BMG labtech). The gain was adjusted to a well containing buffer and DNA but no protein. Curves were fitted with Graphpad Prism and represent the averages of at least three different reactions and two independent protein batches and mean values are plotted with their associated SD.

### Baculoviruses, protein production, and purification

The cDNAs encoding human MAT1 and XPD-FLAG tag were inserted into the transfer vector pAC8_MF under the control of its PH promoter and recombined with baculovirus DNA (Bac10:KO1629 Δ v-cath-Chia) in Sf9 cells to generate the corresponding viruses (V_MAT1 and V_XPD) as described previously^42^. Mouse anti-XPD(2F6), p44(1H5), MAT1(2F5), DsRed, and rabbit anti-MAT1 antibodies were obtained from IGBMC’s facilities.

XPD and core-TFIIH were expressed from baculovirus infected Sf21 suspension cultures as described previously^15,42^. Wild-type CAK and its variants were produced by co-infection using a virus expressing MAT1 (V_MAT1) and a virus for production of the CDK7/Cyclin H complex (V_Cyclin H_CDK7-Strep) with a Strep-tag at the C-terminus of CDK7.

Pellets from infected cells were resuspended in 10 ml of buffer A (20 mM Tris–HCl, pH 8.0, 150 mM KCl, 20% glycerol, 0.1% NP-40, 5 mM β-mercaptoethanol) supplied with Complete protease inhibitor cocktail (Roche^™^). Cells were disrupted using a dounce homogenizer and after centrifugation at 14,000 × *g* for 30 min at 4 °C, the lysate was incubated for 2 h with 200 μl of sepharose beads cross-linked to the M2 anti-Flag antibody (SIGMA^™^) for purification of XPD-FLAG, to the 1H5 anti-p44 antibody for purification of core-IIH or with Streptactin sepharose (IBA^™^) for purification of CAK. After extensive washing in buffer A and equilibration in buffer B (50 mM Tris-HCl pH 8.0, 50 mM KCl, 20% glycerol, 0.01% NP-40, 0.25 mM DTT), proteins were eluted by competition with 2 CV of buffer B containing the appropriate peptide at 1.0 mg/ml for immunoprecipitations or 2.5 mM d-desthiobiotin for elution from Streptactin sepharose (IBA^™^) and quantified.

### Pull-down experiments

Pull-down experiments to evaluate XPD/CAK association were performed in interaction buffer (20 mM Hepes pH 8.0, 100 mM KCl, 10% glycerol EDTA 0.2 mM, 0.05% NP-40, 0.2 mM DTT supplemented with Complete protease inhibitor cocktail (Roche^™^)) using 5 μL of Protein G magnetic beads (Dynadeads^™^) previously loaded with anti-XPD (2F6) for 2 h at 4 °C and saturated with bovine serum albumin (BSA). Following incubation of the magnetic beads for 4 h at 4 °C with a soluble extract prepared from Sf21 cells infected with a virus expressing XPD-Flag or with a control virus expressing the DsRed protein (20 µl in a total volume of 200 µl), beads were successively washed with the interaction buffer containing 100 mM KCl and further incubated with purified CAK wild type or mutant (200 ng in a total volume of 200 µl). Beads were washed with the interaction buffer containing 500 mM KCl and retained complexes analyzed on an sodium dodecyl sulfate (SDS)/PAGE followed by Western blot experiments. Interactions between XPG and XPD variants were analyzed with the same strategy using an anti-XPG antibody (1XPG-1B5).

### In vitro transcription and RNA pol II phosphorylation

Run-off transcription assays were performed using reaction mixes (25 µl) containing the (AdMLP) template (75 ng EcoRI–SalI of the fragment), TFIIB (15 ng), TFIIE (160 ng), TFIIF (500 ng of the phenyl fraction from Pol II/GTF purification scheme), TBP (30 ng), endogenous RNAP II (10 mg of the 1 M DEAE fraction), and a mixture of the purified core-IIH (250 ng), CAK (90 ng) and XPD and its variants (100 and 200 ng, concentrations estimated and adjusted according to the Western blot analysis using the M2 anti-Flag antibody)^15,20^. RNA Pol II phosphorylation was carried out as a runoff transcription except that ATP was added to a final concentration of 4 mM. RNA synthesized by Pol II activity was resolved by SDS/PAGE with 12% (wt/vol) polyacrylamide. Phosphorylation was detected by Western blot using the RNA pol II CTD phospho Ser5 antibody (3EP, ActiveMotif™, 61085).

### Helicase and dual incision assays with human XPD

The helicase activity of hsXPD was analyzed using a 5′ overhang non-labeled substrate corresponding to the translocated strand (5′ TTTTTTTTTTTTTTTTTTTTTTTTTTTCGAGCACCGCTGCGGCTGCACCGGC-3′) and a radiolabeled opposite strand (5′ GCCGGTGCAGCCGCAGCGGTGCTCG-3′). The opposite strand was diluted to 200 nM (10 pmoles in 50 µl) in the presence of 25 mM NaCl and 2.5 mM MgCl_2_, labeled using [γ-^32P^] ATP and T4 polynucleotide kinase (New England BioLabs), purified using Micro Bio-Spin clean-up columns (Bio-Rad) and mixed with a two-fold excess of the translocated strand. The mixture was heated for 2 min at 100 °C and cooled slowly to RT to allow annealing of the DNA heteroduplex.

The strand displacement reaction was performed for 45 min at 30 °C by adding XPD (125 and 250 ng) to an excess of the p34/p44 heterodimer and to the DNA probe at 10 nM in a buffer containing 20 mM Tris-HCl (pH 8.0), 40 mM KCl, 4 mM MgCl_2_, 1 mM DTT, 4 mM ATP, and 0.1 mg/mL BSA. Reactions were stopped by adding 20 mM EDTA, 14% glycerol, 0.2% SDS, and 0.028% bromophenol (10 µL) to the reaction mixture (25 µL). Analyses were performed by migration in a 14% (wt/vol) polyacrylamide gel (acrylamide/bis-acrylamide ratio: 33/1) and autoradiography.

Nucleotide excision repair dual incision assays were performed with wild-type XPD and its variants in a reaction that contains the other TFIIH subunits, XPC-HR23B, XPA, RPA, XPG, and ERCC1-XPF factors and a closed circular plasmid with a single 1,3-intrastrand d(GpTpG) cisplatin-DNA crosslink as a template in a buffer containing 50 mM Hepes-KOH (pH 7.8), 4 mM MgCl_2_, 1 mM DTT, 0.1 mM EDTA, 20% glycerol, 2.5 μg BSA, 50 mM KCl, and 2 mM ATP. Reaction mixes contained XPG (5 ng), XPF/ERCC1 (15 ng), XPC/ hHR23B (10 ng), RPA (50 ng), XPA (25 ng), core-TFIIH (rIIH6) (250 ng), and two quantities of XPD (100 and 200 ng). Reaction mixes (25 µL) were incubated at 30 °C for 90 min and analyzed by electrophoresis followed by autoradiography^15,20^.

### Reporting summary

Further information on research design is available in the Nature Research Reporting Summary linked to this article.

## Supplementary information

Supplementary Information

Reporting Summary

## Data Availability

The coordinates and structure factors for the Arch/MAT1 complex have been deposited in the Protein Data Bank (PDB) under the accession codes 6TUN [doi.org/10.2210/pdb6tun/pdb] The source data underlying Figs. 3c, e–g, 4a–f, 5a–g, and 7c and Supplementary Figs. 2b, c, 3a–d, 4, and 5d, e are provided as a Source Data file. All data are available from the authors upon reasonable request.

## Source Data

Source Data
