# Supplementary material for: In TFIIH the Arch domain of XPD is mechanistically essential for transcription and DNA repair
Source: Nat Commun. 2020 Apr 3;11:1667. doi: 10.1038/s41467-020-15241-9 (PMC7125077; doi:10.1038/s41467-020-15241-9)
Supplement: Supplementary file 1 — Supplementary Information [file 41467_2020_15241_MOESM1_ESM.pdf]

Supplementary information

Supplementary methods:

Thermal unfolding assays

Correct folding of the ctXPD variants was tested by thermal shift assays using sypro orange (Invitrogen) and a qPCR machine (Stratagene mx3005p). The final reaction mix of 25 µl comprised 2 µM ctXPD, 0.1% sypro orange, 20 mM HEPES pH 7.5, 200 mM NaCl, 5 mM $MgCl_2$, and 1 mM TCEP. Unfolding was observed as an increase in fluorescence which was detected at an excitation wavelength of 492 nM and an emission wavelength of 610 nM. Curves were generated with Excel (Microsoft) and melting temperatures represent the average of three different measurements.

Cloning and mutagenesis of ctDDX11.

The ctDDX11 DNA was synthesized by ATG-Biosynthetics (Heidelberg) and subsequently cloned into pETM11 (EMBL, Heidelberg) via SLIC (see main methods) generating pET-DDX11. The KK427/428EE mutation was generated by inverse PCR using KOD Hot Start Polymerase (Novagen) followed by blunt-end ligation using T4 Polynucleotide kinase and T4 DNA ligase (both NE Biolabs) using primers 5'GAGCTGAAAGGTGAAAATCGTATGATGG and 5'TTCGCCAAAGCGCTTCACATACACG.

DNA substrates for ctDDX11.

Helicase substrate:

5'-Cy3- CCATTCCACCCTCTA-3'

5'-TTTTTTTTTTTTTTTTTAGAGGGTGGAATGG –Dab-3'

Expression and purification of ctDDX11

The plasmid encoding wild-type DDX11 or the mutated version were transformed into SoluBL21 (Genlantis) competent cells. All media were supplemented with 50 µg/ml kanamycin. An overnight culture was used to inoculate large-scale expression cultures in TB medium grown at 30°C to an OD_{600} of 0.5 and then induced with 0.4 mM IPTG overnight at 15°C.

The bacterial pellet was resuspended in 50 mM Tris-HCl pH 8.0, 500 mM NaCl, 1 mM TCEP, 10 mM imidazole, 1 EDTA-free protease inhibitor tablet/50 mL cell lysate (Roche), and a few crystals of DNase I. Cells were lysed by two passages through a Microfluidics M-110P microfluidizer at 150 MPa, and centrifuged for 1 h at 60000 x g to remove particulate matter. The clear lysate was loaded on a Histrap FF column (GE Healthcare) pre-equilibrated in 50 mM Tris-HCl pH 8.0, 500 mM NaCl, 1 mM TCEP using an Akta Purifier FPLC system. After loading of the supernatant, the column was washed with 16 column volumes of the same buffer containing 25 mM imidazole, before elution with a gradient from 25 – 250 mM imidazole over 10 column volumes. The protein was then concentrated before loading it on a Superdex 200 26/60 prepgrade (GE Healthcare) column equilibrated in 20 mM HEPES pH 7.0, 200 mM NaCl, 1mM TCEP. The sample was then diluted into 20 mM MES pH 6.5, 1 mM TCEP to a salt concentration of 100 mM NaCl for a final purification step by ion-exchange chromatography. The protein was loaded on a MonoS 10/100 GL column (GE Healthcare) equilibrated in 20 mM MES pH 6.5, 1 mM TCEP, and then eluted with a 100 – 600 mM NaCl gradient over 18 column volumes. The final protein solution was concentrated and buffer exchanged using a spin concentrator into 20 mM HEPES, pH 7.5, 200 mM NaCl, 1mM TCEP and stored at -80°C. The protein concentration was estimated by the A_{280} absorption using an extinction coefficient of 92250 $M^{-1}cm^{-1}$.

ctDDX11 Helicase assays

For helicase assays 2 µM DDX11 were analyzed in the presence of 250 nM DNA and the reaction was started with the addition of 1 mM ATP. As reaction buffer 25 mM HEPES pH 7.5, 25 mM KCl, 1 mM $MgSO_4$, and 1 mM TCEP was used.

ctDDX11 Fluorescence anisotropy

For fluorescence anisotropy 2 nM DNA were used as bait and incubated with the protein for 5min prior to recording. Assays were performed with the same splayed duplex that was used for ctXPD in 25 mM HEPES pH 7.5, 25 mM KCl, 1 mM $MgSO_4$, and 1 mM TCEP.

Supplementary Figures

Supplementary Figure 1

a

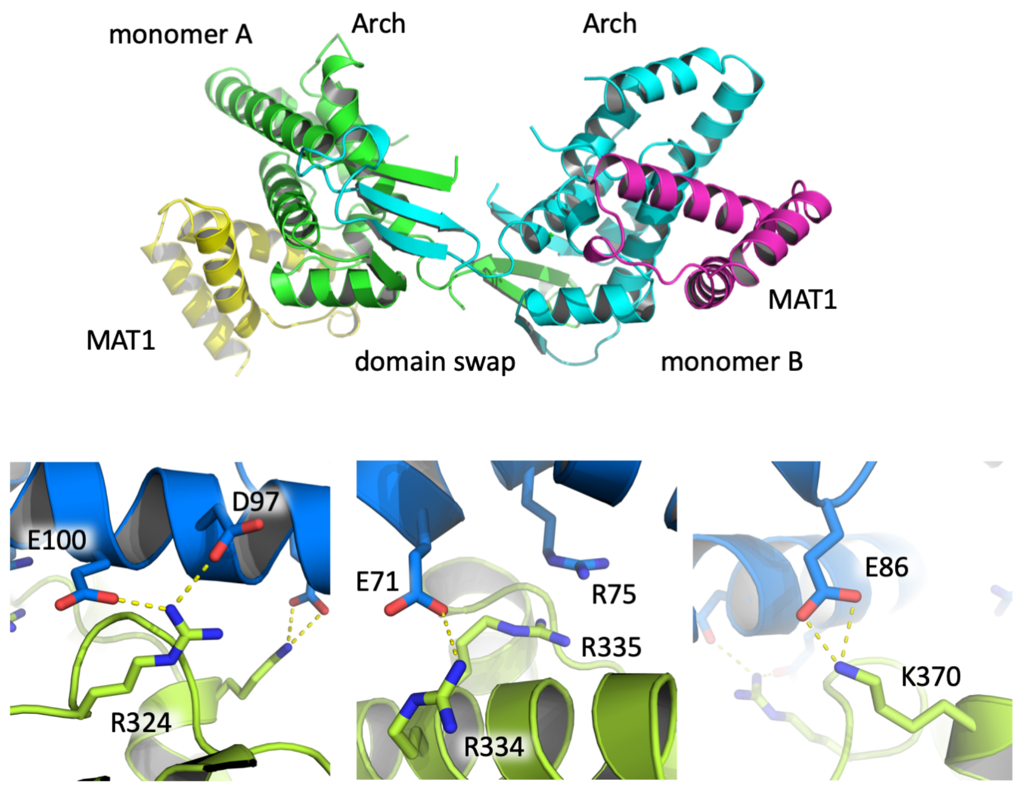

(a) Composition of the asymmetric unit of the Arch/MAT1 complex. The two hetero-dimers in the asymmetric unit are depicted as ribbons. The domain swap is indicated. In monomer A the Arch domain is colored in green and MAT1 in yellow. In monomer B the Arch domain is colored in cyan and MAT1 in hotpink. **(b)** Closeup view on the targeted interactions within the MAT1/Arch domain interface. The Arch domain is colored in green and MAT1 is colored in blue.

Supplementary Figure 2

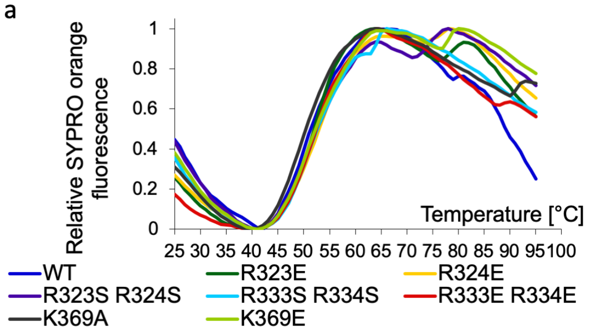

XPD variant	mean T(m)	SD
WT	51.3	1.5
R323E	53.3	1.5
R324E	55.0	1.0
R323S R324S	52.7	2.3
R333S R334S	51.3	0.6
R333E R334E	53.0	0.0
K369A	49.7	0.6
K369E	50.7	0.6

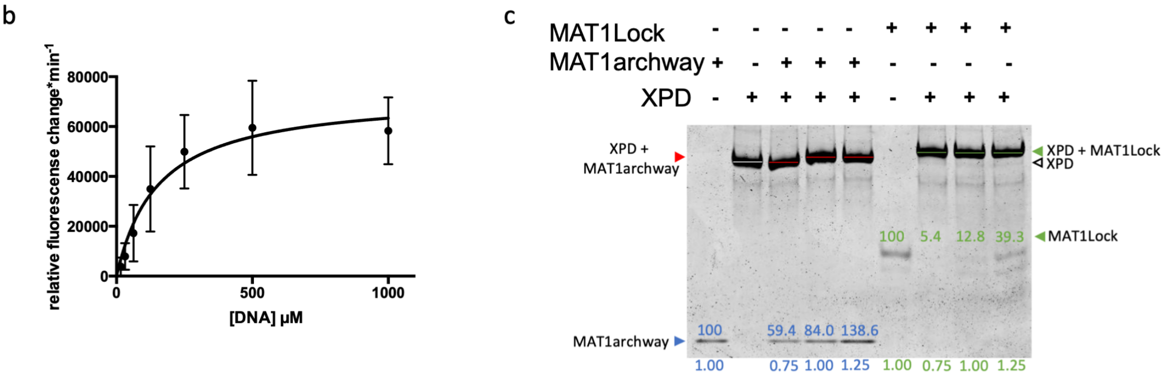

(a) Thermal stability of ctXPD variants. Normalized melting curves of ctXPD variants as determined by differential scanning fluorimetry. The melting points of the different variants are summarized in the Table on the right. **(b)** Helicase activity of ctXPD/ctp44 depending on the DNA concentration. At a DNA concentration of 250 nM DNA ctXPD is fully activated but not oversaturated with DNA. **(c)** Coomassie Blue stained native PAGE analysis of the ctXPD ctMAT1archway interaction and ctXPD ctMAT1Lock interaction. The green and blue numbers below the PAGE denote the molar ratios that were mixed for the interaction study. The blue and green numbers above the bands of the free protein denote the percentage of the unbound ctMAT1 variant.

Supplementary Figure 3

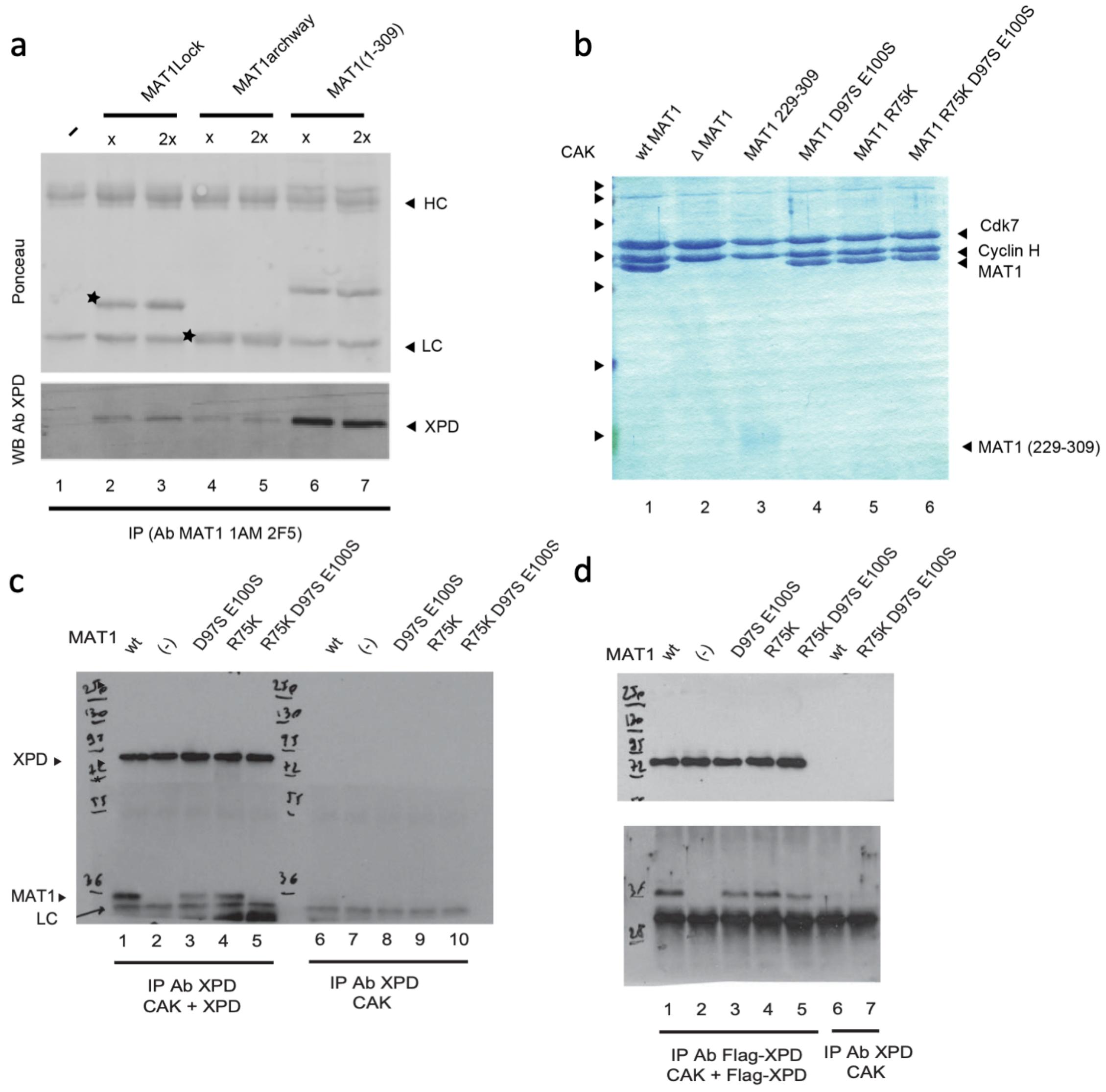

(a) Pull down experiments of human XPD using different MAT1 constructs as bait. The asterisk denotes that these MAT1 variants were produced in *E. coli*. HC and LC are abbreviations for heavy and light chain of the used antibody, respectively. **(b)** Purified CAK complexes harboring different MAT1 variants. **(c)** Original scan of Figure 5A. **(d)** Replicate of Figure 5A performed using an anti-Flag antibody instead of an anti-XPD antibody

Supplementary Figure 4

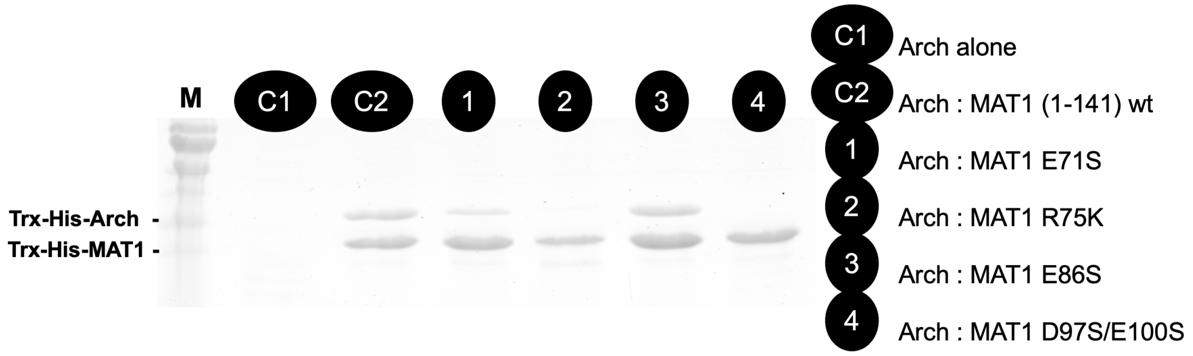

Co-expression analysis of the human Arch/MAT1 complex with different MAT1 variants. Different variants of MAT1Lock (1-141) were used to investigate their ability to solubilize the Arch domain upon co-expression. For expression of the proteins the optimal conditions of the complex as described in the main section were used. Purification was performed using Ni-IDA beads as described in the material and methods section.

Supplementary Figure 5

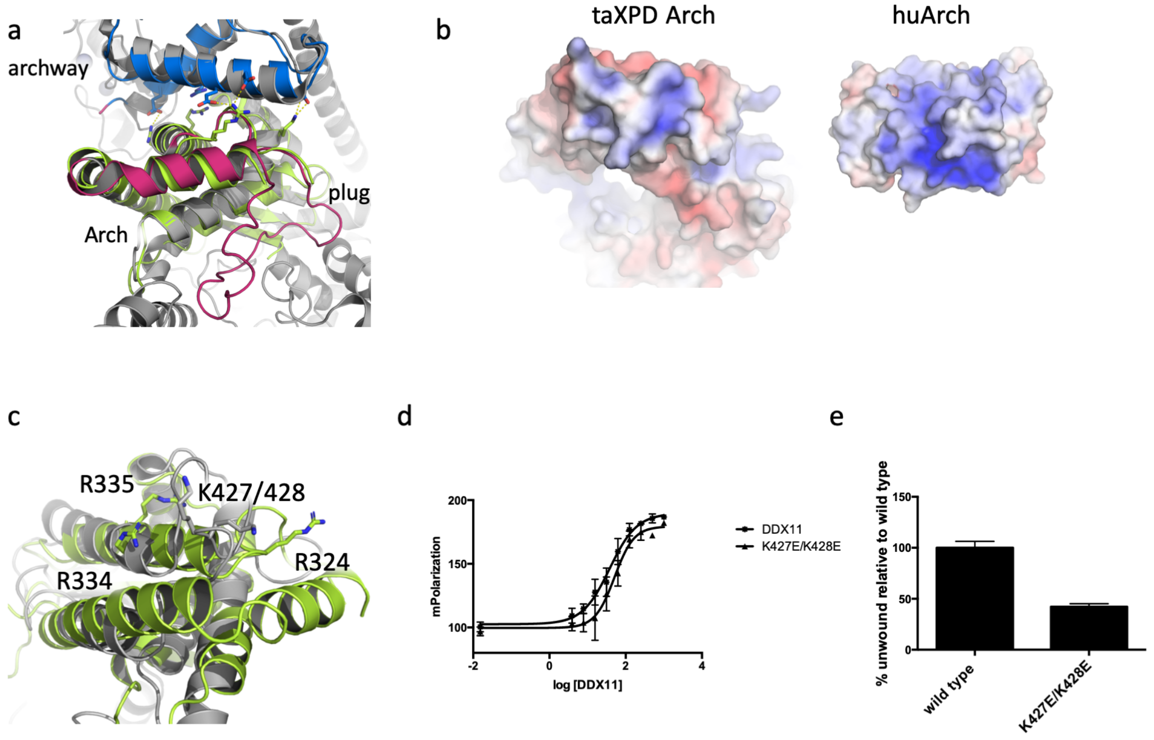

The Arch domain is a functional conserved element. **(a)** Superposition of the TFIIH structure (pdb code 6nmi) and our Arch/MAT1 complex structure indicating the critical residues for the interaction between XPD and MAT1. The plug region is shown in red. **(b)** Surface representation of the Arch domain in archaeal XPD (4a15) and the human Arch domain with their surface potential. **(c)** Superposition of an Arch domain homology model of DDX11 (gray) with the Arch domain of XPD (green). The model was generated using the Swiss-Prot Server (www.expasy.ch). The protein is shown in cartoon mode and selected residues are shown in ball and stick mode. **(d)** DNA binding curves of ctDDX11 and its variants using fluorescence anisotropy. **(e)** Helicase activity of ctDDX11 and its variants.

Supplementary Table 1. Crystallographic Data Collection, Phasing and Refinement Statistics

	**XPD Arch – MAT1 complex (Ir SAD)**	**XPD Arch – MAT1 complex (Native) pdb code 6TUN** [doi.org/10.2210/pdb6tun/pdb]
**Data Collection**		
Space group	$P2_12_12_1$	
Resolution range (Å)	46.07 – 2.25 (2.32-2-25)	46.71 - 2.07 (2.13-2.07)
Cell dimensions a,b,c (Å)	72.58, 78.44, 91.77	74.94, 78.35, 92.33
α, β, γ (°)	90, 90, 90	
Wavelength (Å)	0.8726	1.0064
Observed reflections	333304 (30375)	180766 (13186)
Unique reflections	25556 (2324)	33675 (2591)
R_{meas}	0.246 (1.445)	0.089 (1.001)
$R_{p.i.m}$	0.068 (0.397)	0.038 (0.439)
CC1/2	0.997 (0.827)	0.999 (0.759)
Mean $I/\sigma I$	11.7 (2.4)	11.6 (2.0)
Completeness (%)	100 (100)	99.6 (99.2)
Multiplicity	13.0 (13.1)	5.4 (5.1)
**Phasing**		
Method	SAD	MR
Resolution (Å)	3.0	
Anom. completeness	100 (100)	
Anom. multiplicity	6.9 (6.8)	
No. of anom. scatterers	1	
FOM	0.477	
**Refinement**		
Resolution		46.71 – 2.07 (2.15 – 2.07)
Reflections work/free		32310 / 1286 (3508 / 142)
R_{work} / R_{free}		0.192 / 0.227 (0.292 /0.309)
Number of atoms		
Protein		3972
Ligand/ion		2
Water		151
B factors (Å²)		
Protein		56.4
Ion		56.7
Water		50.3
RMSD		
Bonds (Å)		0.01
Angles (°)		0.88
Ramachandran plot favored / allowed / outliers (%)		97.8 / 2.2 / 0.0
Numbers in parentheses are for the highest resolution shell; SAD: single wavelength anomalous dispersion; MR: molecular replacement; Nat: native; Ir: K2IrCl6 derivatized; FOM: figure of merit after density modification; RMSD: root mean square deviation;		

Supplementary Table 2.

Raw data of helicase activity analysis for human XPD and variants

Helicase

Helicase assays	Exp 1	Exp 2	Exp 3	Exp 1	Exp 2	Exp 3		
	raw data			normalized values			Average	rmsd
blank	8804	6131	2503	0	0	0	0	0
wt	27529	24324	25053	100%	100%	100%	100%	0%
K370A	15127	9750	15953	34%	20%	60%	38%	20%
K370E	11558	7347	3486	15%	7%	4%	9%	5%
K334S/K335S	18423	11483	14574	51%	29%	54%	45%	13%
K334E/K335E	22054	12836	16254	71%	37%	61%	56%	17%
R324S	11198	7454	4840	13%	7%	10%	10%	3%

Supplementary Table 3.

Raw data of transcription activity analysis of human XPD and variants

Transcription

transcription	experiment 1		experiment 2		experiment 1		experiment 2			
	raw data				normalized values					
blank	3057		3474							
	load 1	load 2	load 1	load 2	load 1	load 2	load 1	load 2	average	rmsd
wt	15979	35967	25418	47686	100%	100%	100%	100%	100%	0%
K370E	17025	32690	20721	41368	108%	90%	79%	86%	91%	13%
K370A	14792	27646	22765	40390	91%	75%	88%	83%	84%	7%
R334S/R335S	8523	8520	8006	11887	42%	17%	21%	19%	25%	12%
R334E/R335E	6125	9869	14284	7986	24%	21%	49%	10%	26%	17%
R324S	5233	3911	4581	6200	17%	3%	5%	6%	8%	6%
